# Evaluation of Antibacterial Activity of Thiourea Derivative TD4 against Methicillin-Resistant *Staphylococcus aureus* via Destroying the NAD+/NADH Homeostasis

**DOI:** 10.3390/molecules28073219

**Published:** 2023-04-04

**Authors:** Yachen Hou, Sikai Zhu, Yamiao Chen, Moxi Yu, Yongsheng Liu, Mingkai Li

**Affiliations:** 1 College of Life Sciences, Northwest University, Xi’an 710069, China; 2 Department of Pharmacology, School of Pharmacy, Air Force Medical University, Xi’an 710032, China; 3 Department of Medicinal Chemistry and Pharmaceutical Analysis, School of Pharmacy, Air Force Medical University, Xi’an 710032, China

**Keywords:** thiourea, *Staphylococcus aureus*, drug resistance, antibacterial activity, infection

## Abstract

To develop effective agents to combat bacterial infections, a series of thiourea derivatives (TDs) were prepared and their antibacterial activities were evaluated. Our results showed that **TD4** exerted the most potent antibacterial activity against a number of *Staphylococcus aureus* (*S. aureus*), including the methicillin-resistant *Staphylococcus aureus* (MRSA), *Staphylococcus epidermidis* and *Enterococcus faecalis* strains, with the minimum inhibitory concentration (MIC) at 2–16 µg/mL. It inhibited the MRSA growth curve in a dose-dependent manner and reduced the colony formation unit in 4× MIC within 4 h. Under the transmission electron microscope, **TD4** disrupted the integrity of MRSA cell wall. Additionally, it reduced the infective lesion size and the bacterial number in the MRSA-induced infection tissue of mice and possessed a good drug likeness according to the Lipinski rules. Our results indicate that **TD4** is a potential lead compound for the development of novel antibacterial agent against the MRSA infection.

## 1. Introduction

*Staphylococcus aureus* (*S. aureus*) is a commensal microorganism that mainly resides in skin and mucosa; once it evades host natural defenses, it causes a wide variety of community- and hospital-acquired infections, such as cutaneous abscesses, endocarditis and pleuropulmonary [1,2]. Meanwhile, *S. aureus* can express multiple virulence factors, including hemolysin, leukotoxin, panton-valentine leucocidin and phenol-soluble modulin, allowing it to manipulate the innate and adaptative immune responses, leading to more serious and life-threatening diseases such as toxic shock syndrome [3,4,5]. In additional, *S. aureus* is able to form a complex structure of extracellular polymeric biofilm that protects the bacteria against hostile conditions and antibacterial drugs and causes chronic and/or secondary infections [6,7]. With the wide use of antibiotics, resistant isolates of *S. aureus*, especially methicillin-resistant *Staphylococcus aureus* (MRSA), have occurred, which leads to antibiotic treatment failure and poor clinical outcomes [8,9]. It is estimated that the MRSA form is responsible for around 171,200 healthcare-related infections in Europe every year and double the 30-day mortality compared with its methicillin-sensitive form [10]. Hence, MRSA brought great challenges to world public health due to its high morbidity, mortality and medical costs. The discovery and development of effective antibacterial agents is a central strategy in combating drug resistance. Considering that chemical structure modification of traditional antimicrobial agents cannot completely overcome the mechanism of bacterial drug resistance, especially the variation in bacterial drug targets [11], antibacterial molecules consisting of novel chemical scaffolds represent an important advance in the battle against antibiotic resistance.

Increasing evidence shows that thiourea derivatives play a crucial role in the field of medicinal chemistry and drug development [12]. They exhibit various biological properties, such as anti-inflammatory [13], antitumor [14], antiviral and antibacterial activity [15,16,17,18]. However, the therapeutic efficacy and underlying mechanism of thiourea derivatives to the MRSA-induced infection are uncovered. In the present study, the in vitro antibacterial activity of thiourea derivatives was evaluated, and thiourea derivative (**TD4**) showed the most potent antibacterial activity against MRSA. It reduced the infective lesion size and the bacterial number in the MRSA-induced infection tissue of mice. Additionally, **TD4** disrupted the integrity of MRSA cell wall by destroying the NAD+/NADH homeostasis. This study suggested that **TD4** could serve as a potential candidate for MRSA-induced infection therapy.

## 2. Results

### 2.1. Synthesis of Thiourea Derivatives

**Scheme 1:**
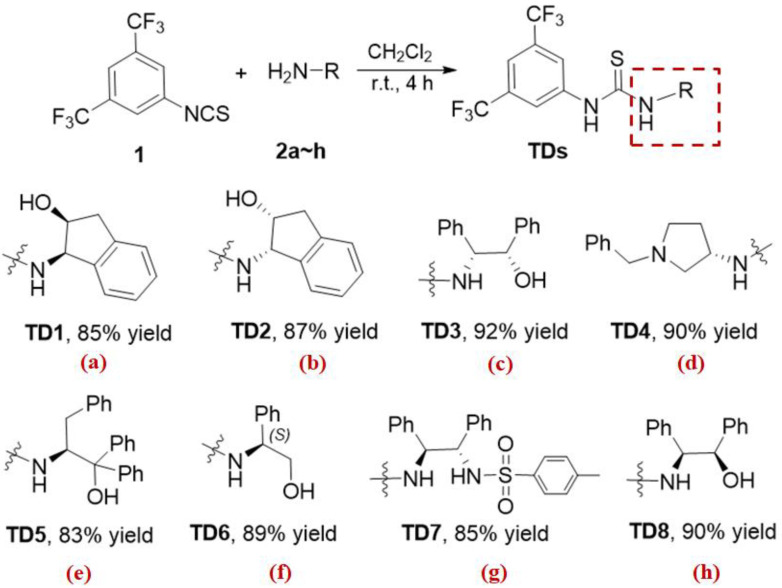
Synthetic route and chemical structures of TDs. (**a**–**h**) the chemical formula of thiourea derivatives.

Thiourea derivatives were prepared in one step by using isothiocyanate 1 and primary amines 2a–h as reactants in dry CH_2_Cl_2_ at room temperature. All TDs were obtained in high yields (Scheme 1). **TD1** and **TD2** were derived from 1-amino-2-indanols 2a and 2b with opposite configurations, respectively [19]. **TD3** and **TD8** were obtained from 2-amino-1,2-diphenylethan-1-ols 2c and 2h with opposite configurations, respectively [20]. L-Phenylalanine-derived bulky vicinal amino alcohol 2e reacts with isothiocyanate 1 to provide **TD5** in 93% yield [21]. **TD6** was generated from L-phenylglycine-derived vicinal amino alcohol 2f and isothiocyanate 1. 1,2-Diphenylethane-1,2-diamine derivative 2g coupled with 1 to afford **TD7** in almost quantitative yield [22]. In particular, **TD4** contains a quite flexible pyrrolidine ring and side chain compared to other TDs, and a pyrrolidine ring was present in many drugs [23]. All prepared TDs were used for inhibition of MRSA with *S. aureus* strains, including methicillin-susceptible *S. aureus* (ATCC 29213) and MRSA (ATCC 43300, USA 300) strains.

### 2.2. Screening and Antimicrobial Spectrum of Thiourea Derivatives

To probe the possible antibacterial activity of thiourea derivatives against MRSA, we study the effect of synthesized compounds to the two tested *S. aureus* strains, and one of the characterized derivates, termed TD4, showed the most potent activity against both methicillin-susceptible *S. aureus* (ATCC 29213) and MRSA (USA 300) strains with MIC at 2 µg/mL (Table 1). Meanwhile, the reference antibiotics oxacillin and ceftazidime did not exert antibacterial activity against MRSA with MIC more than 256 µg/mL. Although the antibacterial activity of thiourea derivatives against *S. aureus* has been reported previously [24,25], the MIC ranged from about 16 to 25 times higher than compound TD4.

In addition, our results showed that the MIC of TD4 against MRSA (ATCC 43300), vancomycin-intermediate-resistant *S. aureus* Mu50, methicillin-resistant *Staphylococcus epidermidis* (MRSE) and *Enterococcus faecalis* (ATCC 29212) were 8 µg/mL, 4 µg/mL, 8 µg/mL and 4 µg/mL. The MIC of TD4 against the hospital-acquired clinical strains MRSA (XJ 26, 216, 317) and vancomycin-resistant *enterococci* (XJ 21, 22, 23) were 8–16 µg/mL. These results indicate that TD4 has potent activity against the Gram-positive bacteria, but it has no obvious antibacterial activity against Gram-negative bacteria with MIC more than 256 µg/mL (Table 2). The selective antibacterial activity is possible due to the difference in cellular structure between the Gram-negative bacteria and Gram-positive bacteria, while the outer membrane of Gram-negative bacteria perform protection against external toxic threats, such as antibiotics [26,27]. Our previous results also showed that the coumarin derivatives exerted antibacterial activity against Gram-positive bacteria selectively, and both coumarin and thiourea derivatives had high liposolubility [28].

### 2.3. Inhibitory Activity of TD4 on MRSA Growth Curves and Bacterial Colonies

**Figure 1:**
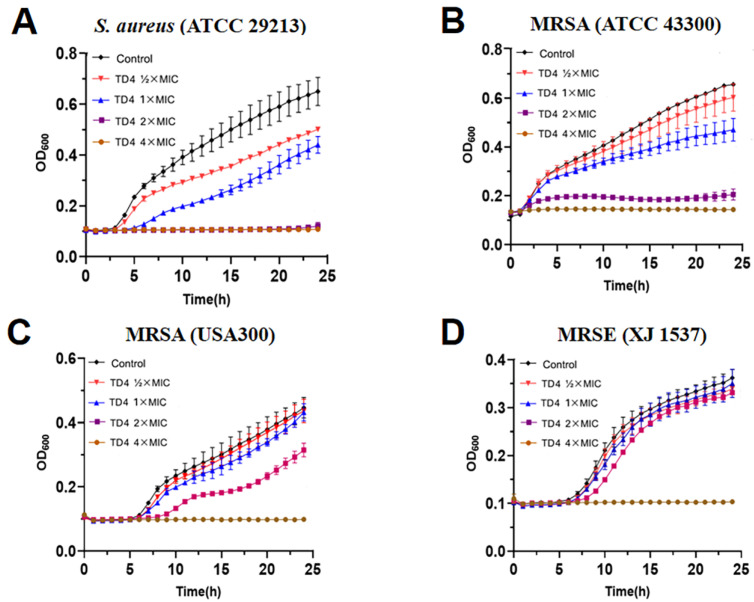
TD4 inhibited the growth curves of (**A**) *S. aureus* (ATCC 29213), (**B**) MRSA (ATCC 43300), (**C**) MRSA (USA 300) and (**D**) MRSE (XJ 1537) in a concentration-dependent manner.

**Figure 2:**
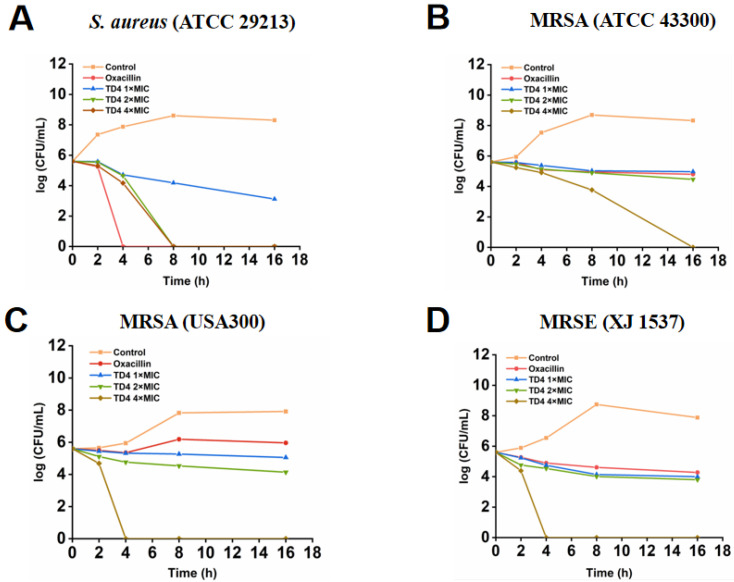
Inhibitory activity of TD4 on bacterial colonies of (**A**) *S. aureus* (ATCC 29213), (**B**) MRSA (ATCC 43300), (**C**) MRSA (USA 300) and (**D**) MRSE (XJ 1537).

Given that compound TD4 inhibits the MRSA strains at the MIC of 4 µg/mL, we next sought to observe the inhibitory activity of TD4 on the dynamic growth curve of MRSA strains. On the *S. aureus* (ATCC 29213), MRSA (ATCC 43300 and USA 300) strains and MRSE (XJ 1537), compound TD4 at 2× and 4× MIC reduced the bacterial number significantly during 24 h culture (Figure 1). Furthermore, in vitro time–kill assay describes the relationship between the concentration of antibacterial agent and the time point of bacteria growth; then, we measured the relationship between the concentration of TD4 and the time point of inhibitory activity on bacterial colonies by time-dependent killing method and the results showed that TD4 could completely inhibit the number of bacterial colonies of *S. aureus* (ATCC 29213) and MRSA (USA 300) strains within 4 h and inhibit the MRSE (XJ 1537) strains within 8 h at 4× MIC concentration. However, TD4 exerts the antibacterial activity to the MRSA (ATCC 43300) within 16 h (Figure 2). The reference antibiotic oxacillin (4 μg/mL) had no obvious antibacterial activity on the drug-resistant strains, including MRSA (ATCC 43300), MRSA (USA 300) and MRSE (XJ 1537). The growth of *S. aureus* showed apparent lag, exponential and stationary phases, and there was no obvious difference between the antibiotic susceptive *S. aureus* strain (ATCC 29213) and MRSA (ATCC 43300 and USA 300); however, drug-resistant bacteria, including MRSA, survive antibiotic treatment, taking advantage of bacterial persistence and slow growth. Our current results suggest that TD4 can in vitro inhibit the growth and bacterial colony formation of MRSA and produce concentration-dependent killing in MRSA efficiently.

### 2.4. Disruption of TD4 on the Integrity of MRSA Cell Wall

**Figure 3:**
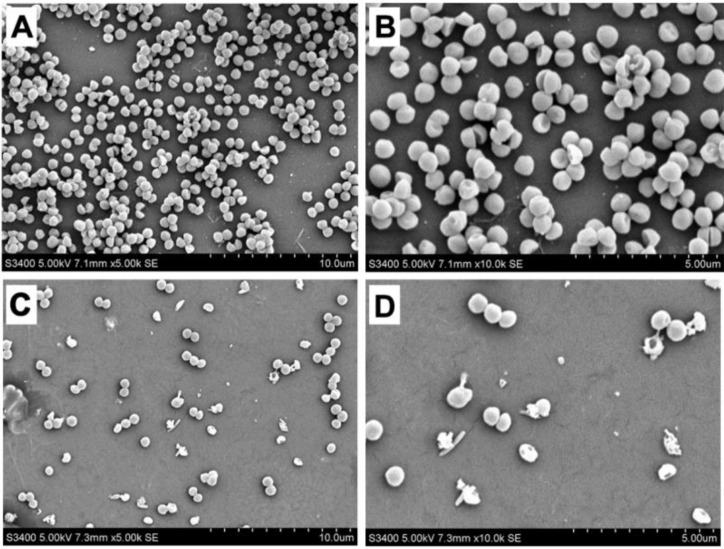
Morphology of MRSA (USA 300) under scanning electron microscopy. (**A**,**B**) show the morphology of USA 300 strain in normal culture at 5000 and 10,000 times magnification, respectively. (**C**,**D**) show the morphology of USA 300 strain treated with 8 μg/mL **TD4** at 5000 and 10,000 times magnification, respectively.

**Figure 4:**
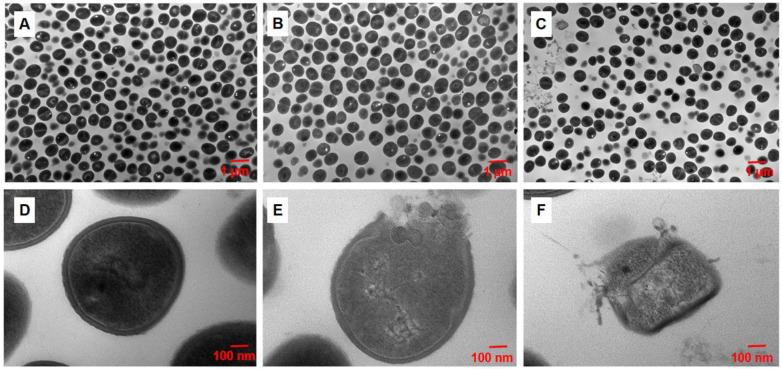
Morphology of MRSA (USA 300) under transmission electron microscopy. (**A**,**D**) show the morphology of USA 300 strain in the control group without compound TD4 treatment, respectively. (**B**,**E**) are the morphology of USA 300 strains treated by 4 μg/mL TD4, respectively. (**C**,**F**) are the morphology of USA 300 strains treated by 8 μg/mL TD4, respectively. (**A**–**C**): scale bar = 1 μm, (**D**–**F**): scale bar = 100 nm.

Bacterial cells are surrounded by the cell wall, which is composed mainly of the peptidoglycan sacculus and prevents bacterial lysis and aids them in coping with diverse environmental challenges [29]. To further explore the possible underlying mechanism of antibacterial activity of TD4, the morphology of MRSA (USA 300) treated by 4 μg/mL and 8 μg/mL TD4 was observed under scanning electron microscope and transmission electron microscope. Consistent with the result of in vitro antibacterial activity assay, the number of bacteria was significantly reduced after TD4 treatment under scanning electron microscopy, the bacterial cell wall was damaged by compound TD4, and there was more MRSA cell debris in the TD4 treatment group compared with the control group (Figure 3). Transmission electron microscopy results showed that compound TD4 disrupted the integrity of the cell membrane of MRSA apparently, and the intracellular content was released from the bacterial cell (Figure 4). These results not only further provide evidence about the antibacterial activity of compound TD4, but indicate that the cell wall structure of Gram-positive bacteria is the possible target of thiourea derivatives.

### 2.5. Therapeutic Efficiency of TD4 on MRSA-Induced Skin Infection

**Figure 5:**
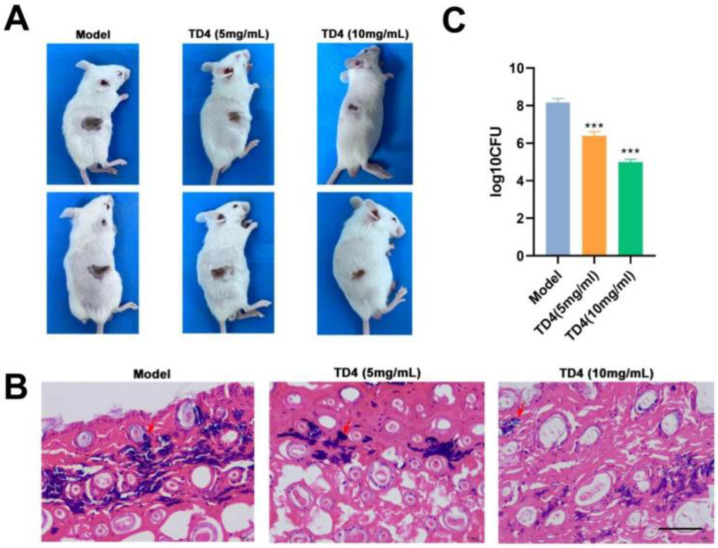
Therapeutic efficiency of TD4 on MRSA-induced skin infections in BALB/c mice. (**A**) The size of infected lesions in mice in the model control group, 5 mg/mL, and 10 mg/mL TD4 groups. (**B**) Skin pathological observation of model control group and intraperitoneal injection of 5 mg/mL and 10 mg/mL TD4 group. Scale bar = 100 μm. (**C**) Analysis of colony-forming units of MRSA USA 300 in infected tissues of mice, *** *p* < 0.001, *n* = 3.

*S. aureus* is the leading cause of skin and soft tissue infections (SSTIs), and MRSA represents a significant burden to the healthcare system [30,31]. Next, we examined the in vivo therapeutic efficiency of compound TD4 in an MRSA-induced skin infection animal model. Compared with the control group treated by vehicle without compound TD4, after intraperitoneal injection of 5 mg/mL and 10 mg/mL TD4 daily for seven days, the area of skin infected lesions and the number of bacteria in infected tissues were reduced significantly. Hematoxylin & Eosin (H.E.) staining showed that TD4 treatment alleviated the pathological changes and reduced the infiltration area of inflammatory cells (Figure 5). These results suggest that TD4 may serve as a potential antibacterial agent for MRSA-induced skin infection treatment.

### 2.6. Cytotoxicity and Drug-Likeness Evaluation of TD4

**Figure 6:**
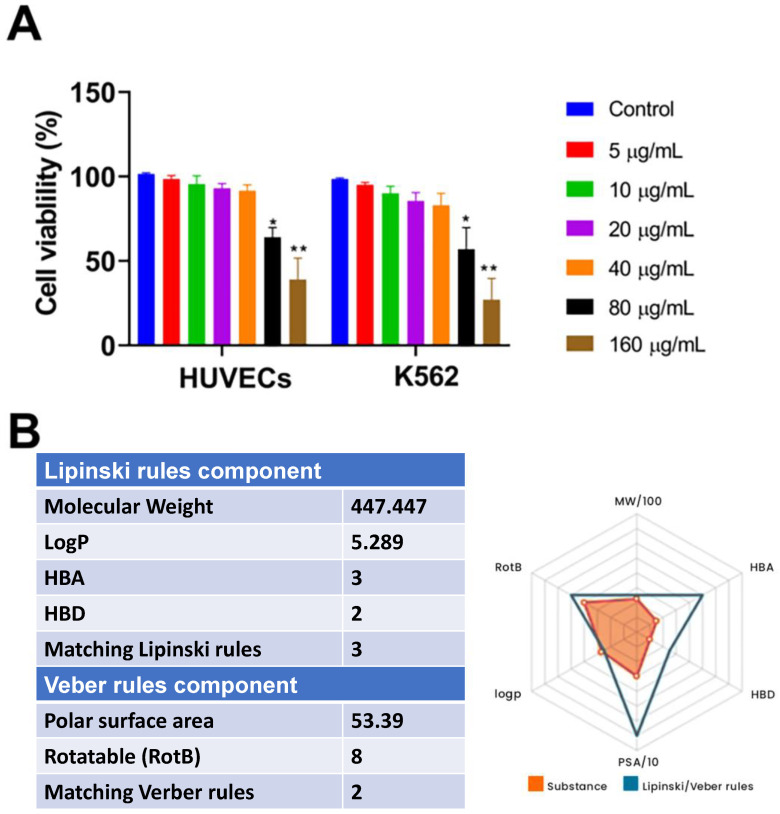
Cytotoxicity (**A**) and drug-likeness (**B**) evaluation of **TD4.** * *p* < 0.05, ** *p* < 0.01, *n* = 3.

Thioureas are the commonly used antithyroid drugs in the clinical setting, such as methylthiouracil, propylthiouracil, thiamzole, and carbimazole, which highlights the druggability of thiourea derivatives as antibacterial agents. Thus, we evaluate the cytotoxicity and drug likeness of compound TD4. As shown in Figure 6A, after being treated with LP4C ranging from 5 to 160 μg/mL for 24 h, in contrast to the cells in the control group, the viability of both human umbilical vein endothelial cells (HUVECs) and human chronic myelogenous leukemia K562 cells was not affected in LP4C treatment groups with a concentration under 40 μg/mL, which was about 10 times higher than the MIC of LP4C against MRSA. The drug likeness of TD4 was evaluated according to the Lipinski rules and Verber rules by a free function on Reaxys (Figure 6B). This estimation indicates that octanol–water partition coefficient (log P) of TD4 is 5.289, it has three hydrogen bond acceptors (HBA), and two hydrogen bond donors (HBD). This result indicates that TD4 possesses a good drug likeness as a potential orally active drug. The easy preparation of TD4 has also provided potential for further pharmacokinetics and toxicological research.

### 2.7. TD4 Disrupted Alanine Dehydrogenase-Dependent NAD+/NADH Homeostasis

**Figure 7:**
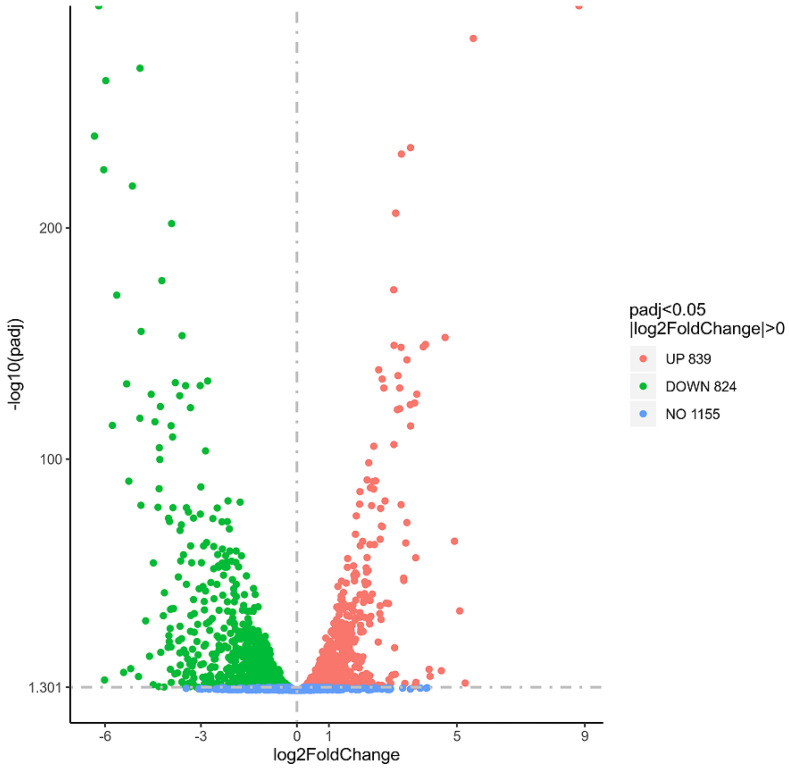
Scatter plot of differentially expressed genes (DEGs) of control vs. treatment group.

**Figure 8:**
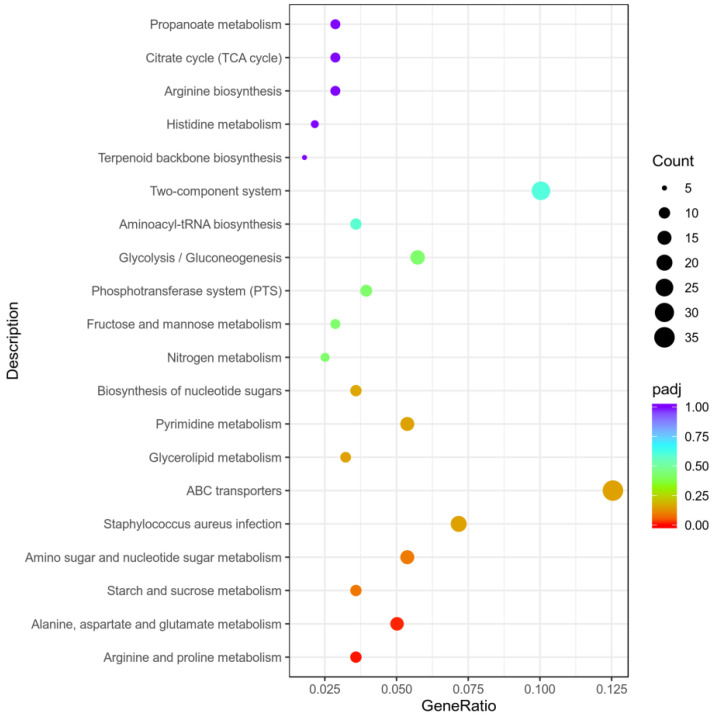
Top 20 statistics of down-regulated pathway enrichment of MRSA (USA 300).

To explore the possible antibacterial mechanisms of TD4, we carried out transcriptome sequencing of bacterial samples treated with vehicle and TD4. The results showed that, among the 2818 detected genes, 1663 genes were expressed significantly in the TD4 treatment group compared with the control group (padj < 0.05), including 839 up-regulated genes and 824 down-regulated genes (Figure 7). Three signal transduction pathways were significantly down-regulated, including glucose metabolism, alanine, aspartic acid, and glutamic acid metabolism, and arginine and proline metabolism (Figure 8).

**Figure 9:**
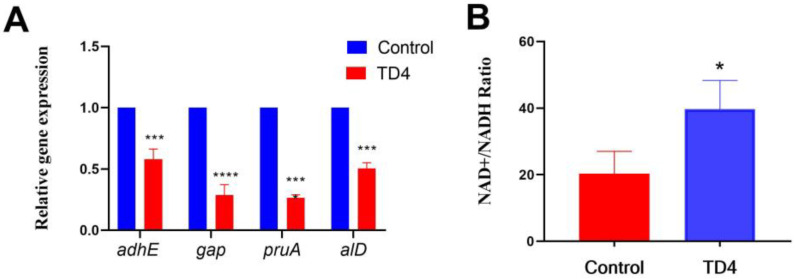
Down-regulated genes and NAD+/NADH ratio after **TD4** treatment. The expression of the *adhE*, *gap*, *pruA*, and *alD* genes after **TD4** treatment was measured by RT-PCR, *** *p* < 0.001, **** *p* < 0.0001 vs. control group, *n* = 3 (**A**). NAD^+^/NADH ratio was measured after **TD4** treatment, * *p* < 0.05 vs. control group, *n* = 3 (**B**).

Among the most differentially expressed genes, the log2 multiples of *adhE*, *alD*, *gap*, and *pruA* were −6.19, −6.03, −3.08, and −2.11 (Table 3), respectively. These genes encode ethanol, acetaldehyde dehydrogenase, alanine dehydrogenase, phosphate dehydrogenase, and tabglutamate semialdehyde dehydrogenase, respectively. Then, real-time PCR results confirmed that TD4 could significantly inhibit the expression of *adhE*, *alD*, *gap*, and *pruA* genes (Figure 9A). Because the down-regulation of *adhE* and *alD* might affect the REDOX reactions of NAD^+^ and NADH and the expression of the adhE gene was correlated with the ratio of NADH to NAD^+^ [32], here, we confirmed that TD4 treatment could inhibit the expression of *adhE* and enhance the ratio of NAD^+^/NADH. These results suggest that TD4 may exert its antibacterial activity by destroying the alanine-dehydrogenase-dependent NAD^+^/NADH homeostasis.

## 3. Materials and Methods

### 3.1. Bacterial Strains, Cells, and Animals

MRSA (USA 300), MRSA (ATCC 43300), and MRSA (Mu 50) strains were purchased from Microbiologics Company (Saint Cloud, MN, USA). *Staphylococcus aureus* (ATCC 29213), *Escherichia coli* (ATCC 25922), *Pseudomonas aeruginosa* (ATCC 27853), *Acinetobacter baumannii* (ATCC 19606), and *Klebsiella pneumonia* (ATCC 700603, ATCC 13885, and ATCC 75297) strains were purchased from the National Antimicrobial Resistance Monitoring Center of China. MRSA (XJ 26, XJ 216, and XJ 317), methicillin-resistant *Staphylococcus epidermidis* (XJ 537), and *Enterococcus faecalis* (XJ21, XJ22, and XJ23) strains were obtained from clinical laboratory of Xijing Hospital (Xi'an, China). Oxacillin and ceftazidime were purchased from China National Institute for Drug and Biological Products Control (Beijing, China). Five-week-old male BALB/C mice were obtained from the Experimental Animal Center of the Fourth Military Medical University. The animal study protocol was approved by the Ethics Committee of the Fourth Military Medical University (protocol code TDLL2018-03-248 and date of 23 February 2018).

### 3.2. Determination of Minimum Inhibitory Concentration

The minimum inhibitory concentration is the lowest concentration of antimicrobial agent that completely inhibits growth of the bacteria determined by Clinical and Laboratory Standards Institute (CLSI) broth microdilution method reported previously [31]. Briefly, a total of 100 μL of bacterial suspension containing 1 × 10^8^ CFU/mL bacteria was added to sterile 96-well microtiter plates. Subsequently, different concentrations (0.25 μg/mL to 256 μg/mL) of test compounds were added into the culture medium, and the plates were incubated at 37 ℃ for 20 h. About 50 μL of 0.2% triphenyl tetrazolium chloride (TTC) was added to each well of microtiter plates and incubated at 37 ℃ for 1.5 h, then measured at the OD_630_ nm value. Compared with the blank control group, the concentration corresponding to the hole with no significant difference is the MIC value of the compound.

### 3.3. Bacterial Growth Curve Assay

The effect of compound TD4 on the growth curve of four bacterial strains, including *S. aureus* (ATCC 29213), MRSA (ATCC 43300), MRSA (USA 300), and MRSE (XJ1537), was measured. The bacteria were inoculated into the culture medium and diluted to 5 × 10^5^ CFU/mL; 150 µL cell suspension was added into the plate wells; then, 150 µL TD4 was added into the suspension to reach the final concentrations (1/2× MIC, 1× MIC, 2× MIC, and 4× MIC). The bacteria were incubated at 37 °C in the automated Bioscreen C system (Labsystems, Helsinki, Finland). The density of the bacterial cell suspensions was measured at 600 nm at 10 min intervals for 24 h, a typical bacterial growth curve that showed the lag phase, exponential phase, stationary phase, and decline phase was plotted, and the effect of TD4 on the characteristic growth pattern of MRSA was measured.

### 3.4. In Vitro Time–Kill Curve Assay

Time–kill curve analyses were performed by culturing MRSA (USA 300) in MH broth medium. Briefly, the compound TD4 was dissolved in DMSO and diluted into 10 mL of MH broth at 1× MIC, 2× MIC, and 4× MIC. Bacteria inoculated in MH broth were set as the control group and growth curves were initially performed to confirm that the bacterial strain could reach a stable early- to mid-log phase after 4 h of preincubation in compound TD4 or antimicrobial-free MH broth medium. The reference antibiotic oxacillin was set as the positive control group. During the incubation at 37 °C, emergent bacterial colonies were counted at 2, 4, 8, and 16 h, the number of bacterial colonies was recorded, and the relationship between the concentration of compound TD4 and the growth rate of MRSA was determined.

### 3.5. Establishment of Bacterial Skin Infection Mouse Model

The subcutaneous infection mouse model was established during *S. aureus* skin infections according to our previous publication [33]. In brief, BALB/c mice were anesthetized through intraperitoneal injection of 10% chloral hydrate at 2.5 mL/kg bodyweight. A rectangular area of approximately 2 × 3 cm was shaved off on the back of mice and the skin was sterilized with 75% alcohol. Bacterial suspension containing MRSA (USA 300) at 1 × 10^8^ CFU/mL was injected into the subcutaneous tissue of mice. After 24 h, 5 or 10 μg/mL compound TD4 was intraperitoneally injected for six consecutive days. The infection lesion size and pathological change was observed and bacterial burden of MRSA from the infection tissue was measured.

### 3.6. Electron Microscopy Observation

MRSA (USA 300) bacteria at 1 × 10^8^ CFU/mL was cultured in MH broth at 120 rpm for 90 min with or without compound TD4 (4 and 8 μg/mL). After that, the cells were harvested and washed by 0.01 M PBS three times and the sample was added to 3% glutaraldehyde for microtome section. After that, the bacterial samples were post-fixed by 1% osmium tetroxide (OsO_4_) for 2 h and dehydrated in 50%, 70%, 80%, 90%, and 95% acetone for 15 min successively. The samples were freeze dried and the images of the samples were observed and recorded under a scanning electron microscope (HITACHI S-3400N, Hitachi, Tokyo, Japan) or transmission electron microscope (JEM-1230, JOEL, Tokyo, Japan).

### 3.7. Cytotoxicity Test

The cytotoxicity of TD4 to the human HUVECs and K562 cells (human umbilical vein endothelial cells (HUVECs) and human CML cell line K562 cells were purchased from ATCC (Manassas, VA, USA)) was examined using the cell counting kit-8 (CCK-8) assay (YEASEN, Shanghai, China). In brief, HUVECs and K562 cells (1 × 10^4^ cells/well) in confluent 96-well cell culture plates were treated with different concentrations of TD4 (5, 10, 20, 40, 80, and 160 μM) for 48 h. Once 10 μL CCK-8 was added into the cell culture medium, the plate was incubated at 37 °C for 2 h. Optical density was measured by the microplate reader (BioTek flx800, Berlin, Germany).

### 3.8. Transcriptome Analysis

MRSA (USA 300) cell pellets in control and TD4 treatment groups were harvested and the total RNA of bacteria was extracted with Trizol reagent and the cleaved RNA fragments were reverse transcribed to generate the sequencing libraries using a gene sequencing system (HiSeq 2000, Illumina, San Diego, CA, USA). The expression of genes between the control group and TD4 treatment group were measured and analyzed as follows: the RNA-seq data were normalized and log-transformed using an oligo R package and multi-array average method, respectively. The significant differences in gene expression were analyzed as Log2 (fold change) ≥1 and a *Padj* value ≤ 0.05. Kyoto Encyclopedia of Genes and Genomes (KEGG) and Gene Ontology (GO) analyses were performed for the mapping of involved pathways.

### 3.9. Real-Time PCR

Total RNA was extracted from MRSA (USA 300) bacteria using RNeasy kit (QIA- GEN, Shanghai, China), and primer sequences are listed as follows (adhE, forward: TCATTGCACTTGGTGGTGGT, reverse: GAACGTCGCATTTTCAGGCA; gap, forward: AGGCTATGGGGCAAAGGTTA, reverse: CGCCAACTGGTACGATGACT; alD, forward: TGTAGCTTGCACACCCGAAA, reverse: CAGCTTCCCATGCTTGTTCG; pruA, forward: GCTACACCATCAACAGATACAGC, reverse: ACGCCAAATGCTCCACCTAA; gyrB, forward: ACAGGAATCGGTGGCGACTTTG, reverse: GCTCCATCCACATCGGCATCAG). Isolated RNA was quantified and reverse transcribed into cDNA using PrimeScript RT. RT-PCR was performed using the Takara Bio.prex Taq RT-PCR system Co., Kyoto, Japan, as follows: cDNA was denatured at 95 °C for 30 s and amplified for 40 cycles 95 °C for 10 s, 60 °C for 31 s, 95 °C for 15 s, 60 °C for 1 min, and 95 °C for 15 s. The 2^−∆∆^Ct method was used for comparative analysis with the control group.

### 3.10. NAD^+^/NADH Ratio Measurement

The two pyridine nucleotides were extracted from a culture MRSA (USA 300) sample by a modification of the protocol described previously, and the levels of NAD^+^ and NADH were measured by the cycling assay [34]. Briefly, 50 mM NaOH/1 mM EDTA solution was added to the harvest MRSA cell pellet, after vortex and sonication in an ice-cold water bath; then, it was incubated at 80 °C for 10 min and cooled on ice for 2 min. Samples were neutralized with 1 mM HCl and 500 mM KH_2_PO_4_ at pH 5.5, then centrifuged for 10 min at 4 °C; the supernatant was transferred to a new tube, the volume was record, and the cycling assay was performed.

### 3.11. Statistical Analysis

The results are expressed as mean ± standard deviation. One-way and two-way analysis of variance (ANOVA) was used for statistical evaluations, with a probability value of *p* < 0.05 considered indicative of statistical significance.

## 4. Conclusions

In this study, we synthesized a series of thiourea derivatives and observed their antibacterial activity against drug-susceptive *S. aureus* and MRSA; the compound TD4 not only showed the most potent in vitro antibacterial activity, but exerted in vivo therapeutic efficiency in MRSA-induced skin and soft tissue infection. Furthermore, TD4 could disrupt NAD^+^/NADH homeostasis and integrity of the bacterial cell wall. These results indicate that thiourea derivative TD4 is a potential antibacterial agent for MRSA infection therapy.

## Figures and Tables

**Table 1:** Antibacterial activity of thiourea derivatives.

Compound	MIC(µg/mL)	
	*S. aureus* (ATCC 29213)	MRSA (USA 300)
TD1	4	8
TD2	8	8
TD3	2	16
TD4	2	4
TD5	4	32
TD6	32	32
TD7	16	16
TD8	8	16
Oxacillin	2	>256
Ceftazidime	4	>256

**Table 2:** Antimicrobial spectrum of TD4.

Microorganism	MIC (µg/mL)
G^+^ Bacteria	
*Staphylococcus aureus* (MSSA, ATCC 29213)	2
*Staphylococcus aureus* (MRSA, ATCC 43300)	8
*Staphylococcus aureus* (MRSA, VISA, Mu50)	4
*Staphylococcus aureus* (MRSA, USA 300)	4
*Staphylococcus aureus* (MRSA, clinical strain XJ 26)	8–16
*Staphylococcus aureus* (MRSA, clinical strain XJ 216)	8–16
*Staphylococcus aureus* (MRSA, clinical strain XJ 317)	8–16
*Methicillin-resistant Staphycoccus epidermidis* (clinical strain XJ 1537)	8
*Enterococcus faecalis* (ATCC 29212)	4
*Vancomycin resistant Enterococcus* (clinical strain XJ 21)	8–16
*Vancomycin resistant Enterococcus* (clinical strain XJ 22)	8–16
*Vancomycin resistant Enterococcus* (clinical strain XJ 23)	8–16
G^−^ Bacteria	
*Escherichia coli* (ATCC 25922)	>256
*Multi-drug resistant clinical strain* (XJ 74283)	>256
*Multi-drug resistant (*SL 1344)	>256
*Acinetobacter baumannii* (ATCC 19606)	>256
*Pseudomonas aeruginosa* (ATCC 27853)	>256
*Multi-drug resistant clinical strain* (XJ 75315)	>256
*Klebsiella pneumoniae* (ATCC 700603)	>256
*Klebsiella pneumoniae* (ATCC 13885)	>256
*Multi-drug resistant clinical* (ATCC 75297)	>256

**Table 3:** Log2 values of significantly down-regulated genes.

Gene ID	Gene Name	log_2_Fold Change
USA 300_RS00795	*adhE*	−6.19
USA 300_RS07360	*alD*	−6.03
USA 300_RS13865	*pruA*	−2.11
USA 300_RS08940	*gap*	−3.80

## Data Availability

Data are contained within the article.
